# A validation study using a modified version of Postural Assessment Scale for Stroke Patients: Postural Stroke Study in Gothenburg (POSTGOT)

**DOI:** 10.1186/1743-0003-8-57

**Published:** 2011-10-06

**Authors:** Carina U Persson, Per-Olof Hansson, Anna Danielsson, Katharina S Sunnerhagen

**Affiliations:** 1 The Institute of Neuroscience and Physiology, Sahlgrenska Academy at the University of Gothenburg, Gothenburg, Sweden; 2 The Institute of Medicine, Sahlgrenska Academy at the University of Gothenburg, Göteborg, Sweden; 3 Sunnaas Rehabilitation Hospital and Medical Faculty, Oslo University, Oslo, Norway

## Abstract

**Background:**

A modified version of Postural Assessment Scale for Stroke Patients (PASS) was created with some changes in the description of the items and clarifications in the manual (*e.g. much help was defined as support from 2 persons*). The aim of this validation study was to assess intrarater and interrater reliability using this modified version of PASS, at a stroke unit, for patients in the acute phase after their first event of stroke.

**Methods:**

In the intrarater reliability study 114 patients and in the interrater reliability study 15 patients were examined twice with the test within one to 24 hours in the first week after stroke. Spearman's rank correlation, Kappa coefficients, Percentage Agreement and the newer rank-invariant methods; Relative Position, Relative Concentration and Relative rank Variance were used for the statistical analysis.

**Results:**

For the intrarater reliability Spearman's rank correlations were 0.88-0.98 and k were 0.70-0.93 for the individual items. Small, statistically significant, differences were found for two items regarding Relative Position and for one item regarding Relative Concentration. There was no Relative rank Variance for any single item.

For the interrater reliability, Spearman's rank correlations were 0.77-0.99 for individual items. For some items there was a possible, even if not proved, reliability problem regarding Relative Position and Relative Concentration. There was no Relative rank Variance for the single items, except for a small Relative rank Variance for one item.

**Conclusions:**

The high intrarater and interrater reliability shown for the modified Postural Assessment Scale for Stroke Patients, the Swedish version of Postural Assessment Scale for Stroke Patients, with traditional and newer statistical analyses, particularly for assessments performed by the same rater, support the use of the Swedish version of Postural Assessment Scale for Stroke Patients, in the acute stage after stroke both in clinical and research settings. In addition, the Swedish version of Postural Assessment Scale for Stroke Patients was easy to apply and fast to administer in clinic.

## Background

The ability to maintain postural balance is often reduced for patients who have suffered a stroke [1-3]. Reliable measurements, designed to assess and monitor postural balance in the initial phase after stroke, are needed for prognostic identification, adequate reporting between different caregivers and to evaluate training effects throughout the rehabilitation process. There is no consensus which of several different test to use in clinical practice, which is an indication that there still is no perfect measure. The Postural Assessment Scale for Stroke Patients (PASS) [4] is developed specifically for stroke patients and has shown to have high interrater and intrarater reliability [4] good individual item agreement [5], acceptable test-retest reliability [6] and high test-retest reliability [7]. The PASS examines the patient's ability to maintain or change a given lying, sitting or standing posture, is easy to handle in the clinic and applicable to all patients, even those with very poor postural performance [4]. Despite the good qualities described for the PASS, we noted in our clinical practice a need for some modifications and clarifications. However, to be applicable in clinical and research settings this modification of the PASS required subsequent validation regarding reliability. Furthermore, regarding the ordinal nature of data in PASS validation should be performed with a statistical analysis aimed for calculations of non-parametric data, besides traditional statistical methods. As far as we know, newer statistical analysis such as rank- invariant method has not been used to explore the PASS before. The aim of this study was to assess the intrarater and the interrater reliability for the grading of postural balance using a modified version of the PASS, in patients at a stroke unit, in the acute phase after their first event of stroke.

## Methods

A modified version of PASS, the Swedish version of PASS (SwePASS), was created with some changes in the description of the items and clarifications in the manual (Additional File 1). The modifications were done after needs perceived during clinical use of the scale. In the SwePASS, "much help" is defined as "support from two persons" and "little help" is defined as "support from one person". The expression "...support from 1 person" and "support from 2 persons" are daily used in clinic, while we found the terms "..little help" or "..much help" not clear enough. In the SwePASS' manual it is specified that the patients' feet should be supported on the floor in item 4 "Sitting without support". "Arm movements above the shoulder level" was a definition too vague to use in a scale aimed to be used repetitively over time, where it is basic that the same movements are measured from time to time. Therefore, item 7 "Standing without support", with "arm movement above the shoulder level" is in the SwePASS specified as in standing performing the task "*Draw hand/s from forehead to neck *(like drawing fingers throw the hair) *altered with the arms hanging parallel with the trunk to avoid tiredness*". Item 10 "Standing picking up an *object/pencil*" is changed and defined as "Standing picking up *a shoe*" with the ambition to minimize possible impact of fine motor skills. The original developers of the PASS have accepted the final version of the SwePASS.

A translation into Swedish was performed merging the French original version, supplied by the authors, and their English version published in 1999 [4]. "Forward-backward-translation" was performed as recommended by Streiner and Norman [8]. The modified PASS (SwePASS), as the original PASS, comprises 12 items, ordinal scored from 0 to 3, with a maximal sum score of 36. In the manual for the SwePASS, unlike the original PASS, the items are listed in the same order as they are logically performed in clinical use.

### Study populations

The intrarater and interrater reliability investigations were performed at the same stroke unit, but during different time periods. The inclusion criterion for both studies was first-ever stroke, defined according to the World Health Organisation criteria [9]. Exclusion criteria were co-morbidities that could interfere with postural control or ability to cooperate in the assessment situations, e.g. leg amputation, diagnosis of dementia or severe psychiatric diseases. At the time of inclusion, demographic and medical data were gathered from the patients' charts. The ethics committee at the University of Gothenburg approved the studies and written informed consent was obtained. If the patient was not able to understand the information, the next of kin gave informed consent.

### Methods

To describe the study population, the clinical physiotherapist carried out assessments using the Modified Motor Assessment Scale Uppsala Akademiska Sjukhus-95 (MAS UAS-95) [10] and the Berg Balance Scale (BBS) [11,12] according to clinical routine.

All SwePASS assessments were done by physiotherapists, not involved in the patients' rehabilitation, who were previously instructed by one of the authors (C.U. P.), how to perform the assessments and in which order (Additional File 1). For both the intrarater and the interrater reliability investigation, the patients were assessed with the SwePASS twice within a 24 hour interval between days four and seven after the stroke onset. All assessments were performed bedside on the ward (with the bed in the lowest position, allowing support for the patient's feet), not with an examination table, like a Bobath plane as described in the original PASS [4]. In the intrarater reliability study, the same physiotherapist assessed the patients on both occasions. Between the two occasions the physiotherapists treated other patients (outside the study). Over the study period, 5 physiotherapists were involved. For the interrater reliability study, the same 2 raters, in a randomised order, carried out the assessments. To minimize recall bias, the physiotherapist did not have access to the previous test protocol.

### Statistics

All analyses were performed using the Statistical Package for Social Services (SPSS^©^) computer program (Version 17 SPSS Inc., Chicago, IL). The level of significance used was p < 0.05. Both the intrarater and the interrater reliability were tested using paired assessments, item for item. The Spearman's rank correlation coefficient (*r*_s_) identifies the strength of correlation within a data set of two variables, and whether the correlation is positive or negative. For evaluation of the correlation we used Currier's definition [13]; ≤0.69 = poor, 0.7-0.79 = fair, 0.80-0.89 = good and 0.90-0.99 = high correlation. The Kappa coefficient (k) identifies the strength of agreement, where a value of 1 implies perfect agreement. For additional evaluation of agreement, we used Fayers' guideline values of k to indicate the strength of agreement [14]; < 0.2 = poor, 0.21-0.40 = slight, 0.41-0.60 = moderate, 0.61-0.80 = good and 0.81-1.00 = very high agreement. For calculation of Percentage Agreement (PA), which was used both for intra- and interrater reliability, we used the formula (agreements/(agreements + disagreements)) * 100 = P% [15]. PA measures exact agreement (diagonal). Additionally, due to this fact and to the ordinal nature of data, the rank-invariant method for inter-scale comparison, described by E Svensson, was applied [16,17]. This method estimates systematic differences between raters; Relative Position (RP) and systematic differences in concentrations of the score chosen, Relative Concentration (RC). E Svensson's method makes it possible to identify and measure systematic disagreement related to the group, RP and RC, separately from disagreement caused by individual variability, Relative rank Variance (RV). RP and RC can be reduced or taken into account when the reason for such a systematic disagreement is present. However, RV, which is a measure of non-systematic variance, cannot be explained by the behaviour of the scale or the raters. The values for RP and RC range from (-1) to 1 and the values for RV range from 0 to 1. A RP or RC value of 0 means that there are no systematic changes, while a value of 1/-1 means that there are systematic differences. RV is hard to interpret, but RV < 0.1 would in general be regarded as negligible [17].

## Results

Initially we recruited 116 patients to the intrarater reliability study. However, the analyses were based on 114 patients, since 1 patient was excluded due to missing data (SwePASS) and further one patient dropped out just before the second test occasion. Beyond this, there were not any drop outs or deaths from inclusion until the finish of the reliability studies. Table 1 provides the participant characteristics of the 114 patients in the intrarater reliability study, and of the 15 patients included in the interrater reliability study. Incomplete test data (12 for the M-MAS UAS-95), or missing data (4 for the M-MAS UAS-95 and 3 for the BBS) were excluded from the analysis.

**Table 1:** Participant characteristics for the study populations in the two reliability investigations

	Intrarater	Interrater
	reliability	reliability
	n 114	n 15
Age, years		
Mean (SD)	73.85 (10.88)	76.7 (7.8)
Median (range)	74.41 (42-94)	79 (44-90)
Gender, *n *(%)		
Male	67 (59%)	8 (53%)
Female	47 (41%)	7 (47%)
Stroke type, *n *(%)		
Ischemic	111 (97%)	15 (100%)
Haemorrhagic	13 (3%)	
Side location of the stroke		
Left	60 (53%)	8 (53%)
Right	54 (47%)	7 (47%)
M-MAS UAS-95 median (range) score 0-55 (n 98)	47 (0-55)	38 (19-55)
BBS median (range) score 0-56 (n 111)	39 (0-56)	39 (0-56)
SwePASS 1st occasion median (range) score 0-36	30 (3-36)	30 (10-36)
SwePASS 2nd occasion time median (range) score 0-36	30 (3-36)	31 (8-36)

M-MAS UAS-95: Modified Motor Assessment Scale Uppsala Akademiska Sjukhus;

BBS: Berg Balance Scale; SwePASS: Swedish version of Postural Assessment Scale for Stroke Patients.

### Intrarater reliability

The assessments using the SwePASS were at median performed on the 5^th ^day post stroke (range 4-7 days). The mean time between the first and second assessments was 2.3 hours (median 1.0 hours, range 1-23 hours), with about 66% of the patients assessed with 1 hour difference and about 95% of the patients assessed with 4 hours difference or less.

Table 2 demonstrates the Spearman's rank correlation coefficient (*r*_s_), the Kappa coefficient (k) and the Percentage Agreement (PA), for each item of SwePASS in the intrarater study. The *r*_s _were high (in line with adopting Currier's guideline values) to indicate the strength of agreement [13] for all but three single items. For the three items that differed (1, 3 and 8) *r*_s _was in the upper limit of a good correlation. In evaluation of k, 9 of the items showed very high and two demonstrated good agreement according to Fayers' definition of strength of agreement (14). The lowest PA was found for item 8 and 9, while the remaining ten items showed a PA value of 94% or more.

**Table 2:** Intrarater reliability; Spearman's (r_s_), the Kappa coefficient (k) and the Percentage Agreement (PA) (n 114)

Item	Spearman's	*p*	Kappa	*p*	PA
	(r_s_)		(k)		
1. Supine to affected side lateral	0.88	< 0.001	0.86	< 0.0001	96%
2. Supine to non affected side lateral	0.98	< 0.001	0.93	< 0.0001	97%
3. Supine to sitting up on edge of bed	0.89	< 0.001	0.85	< 0.0001	95%
4. Sitting without support	0.93	< 0.001	n a	n a	97%
5. Sitting to standing up	0.95	< 0.001	0.91	< 0.0001	94%
6. Standing with support	0.94	< 0.001	0.91	< 0.0001	96%
7. Standing without support	0.95	< 0.001	0.88	< 0.0001	94%
8. Standing on non paretic leg	0.88	< 0.001	0.70	< 0.0001	82%
9. Standing on paretic leg	0.92	< 0.001	0.75	< 0.0001	86%
10. Standing, picking up a shoe from the floor	0.96	< 0.001	0.89	< 0.0001	94%
11. Sitting down from standing up	0.93	< 0.001	0.85	< 0.0001	94%
12. Sitting on edge of bed to supine	0.92	< 0.001	0.84	< 0.0001	94%

SwePASS: Swedish version of Postural Assessment Scale for Stroke Patients;

n a: not applicable, Kappa statistics could not be computed, since they require a symmetric 2-way table in which the values of the first variable match the values of the second variable.

The distribution of received scores from the two assessment occasions using the SwePASS is presented in Table 3. There is a pattern, with the majority of the patients being given the scores 2 or 3 in all items except in items 8 and 9. In contrast, in these two items most patients were given the score 0. For items 2 and 3 there was no floor effect since no patient received the lowest score.

**Table 3:** Intrarater reliability; the distribution of scores from first and second test occasion (n 114)

	Item																							
	1		2		3		4		5		6		7		8		9		10		11		12	
	Test occasion																							
	1	2	1	2	1	2	1	2	1	2	1	2	1	2	1	2	1	2	1	2	1	2	1	2

Score	Number of patients at each score level																							

0	1	1	0	0	0	0	10	11	3	3	5	5	25	25	70	65	71	71	18	21	4	4	2	3
1	3	3	6	6	7	8	1	0	11	13	9	10	3	3	10	13	15	11	7	4	11	10	10	9
2	14	10	19	18	17	17	2	2	22	17	12	12	12	7	10	8	8	7	18	17	14	13	15	15
3	96	100	89	90	90	89	101	101	78	81	88	87	74	79	24	28	20	25	71	72	85	87	87	87

Table 4 supplies the results of Relative Position (RV), Relative Concentration (RC) and Relative rank Variance (RV) for the intrarater reliability. Along with RP, item 1 and 7 have statistically significant but small differences. Corresponding to the estimates of RC, item 7 shows a statistically significant but small difference in concentration of score chosen. In these results, no RV was present.

**Table 4:** Intrarater reliability;Relative Position (RP), Relative Concentration (RC) and Relative rank Variation (RV) (n 114)

		95% CI			95% CI			95% CI	
Item	RP	Lower	Upper	RC	Lower	Upper	RV	Lower	Upper
1. Supine to affected side lateral	0.03	0.001	0.066	-0.01	-0.030	0.000	0.00	0.0000	<0.0001
2. Supine to non affected side lateral	0.01	-0.001	0.025	-0.00	-0.030	0.030	0.00	0.0000	<0.0001
3. Supine to sitting up on edge of bed	-0.01	-0.046	0.026	-0.01	-0.030	0.020	0.00	0.0000	0.0004
4. Sitting without support	-0.00	-0.023	0.021	-0.01	-0.040	0.020	0.00	0.0000	<0.0001
5. Sitting to standing up	0.02	-0.009	0.045	-0.04	-0.070	0.000	0.00	0.0000	<0.0001
6. Standing with support	-0.01	-0.035	0.017	-0.001	-0.030	0.020	0.00	0.0000	0.0001
7. Standing without support	0.03	0.004	0.062	-0.05	-0.090	-0.010	0.00	0.0000	<0.0001
8. Standing on the non paretic leg	0.04	-0.000	0.089	0.00	-0.070	0.080	0.00	0.0000	0.0072
9. Standing on the paretic leg	0.02	-0.019	0.055	-0.06	-0.110	0.000	0.00	0.0000	0.0108
10. Standing, picking up a shoe from the floor	0.00	-0.024	0.026	-0.03	-0.070	0.010	0.00	0.0000	0.0004
11. Sitting down from standing up	0.02	-0.013	0.048	-0.00	-0.040	0.030	0.00	0.0000	0.0002
12. Sitting on edge of bed to supine	-0.00	-0.032	0.030	-0.00	-0.040	0.030	0.00	0.0000	0.0002

CI: Confidence Interval

### Interrater reliability

The assessments using SwePASS were at median performed on the 5^th ^day post stroke (range 4-7 days) (as for the intrarater reliability study). The mean time, between the first and second assessments was 4.0 hours (median time 1.7, range 1-23 hours) with almost 7% being assessed with a time between the assessments of 1 hour and around 93% being assessed with 7.5 hours or less between the assessments. The mean and median time required to administer SwePASS, registered only in the interrater reliability study, was 8 minutes for both the first and the second assessment.

Table 5 shows the results of *r*_s _and PA for the interrater reliability. Three quarters of the single items were identified as having high *r*_s_, according to Currier's definition [13]. There is a pattern with lower *r*_s _in the items including standing in the interrater test (Table 5) compared with the intrarater test (Table 2). Concerning PA, item 5 ("Sitting to standing up") had the highest PA, while in contrast item 8 ("Standing on non paretic leg") had the lowest PA.

**Table 5:** Interrater reliability; Spearman's (r_s_) and Percentage Agreement (PA) using the SwePASS (n 15)

Item	Spearman's	*p*	PA
	(r_s_)		
1. Supine to affected side lateral	0.85	< 0.001	87%
2. Supine to non affected side lateral	0.90	< 0.001	80%
3. Supine to sitting up on edge of bed	0.97	< 0.001	87%
4. Sitting without support	0.99	< 0.001	87%
5. Sitting to standing up	0.95	< 0.001	93%
6. Standing with support	0.93	< 0.001	87%
7. Standing without support	0.86	< 0.001	87%
8. Standing on non paretic leg	0.77	0.001	67%
9. Standing on paretic leg	0.84	< 0.001	73%
10. Standing, picking up a shoe from the floor	0.94	< 0.001	87%
11. Sitting down from standing up	0.96	< 0.001	87%
12. Sitting on edge of bed to supine	0.93	< 0.001	87%

In Table 6, the results of RV, RC and RV for the interrater reliability study are exposed. According to the estimates of RP, there are no statistically significant differences between raters. Nevertheless, items 2 and 9 differ, with higher values of RP. RC shows that there are no statistically significant differences in concentrations of the score chosen. However, three items (1, 3 and 4) diverged with higher RC. According to RV, nothing indicated that there was any non-systematic variance for the single items, except for a small RV for item 8.

**Table 6:** Interrater reliability; Relative Position (RP), Relative Concentration (RC) and Relative rank Variation (RV) (n 15)

		95% CI			95% CI			95% CI	
Item	RP	Lower	Upper	RC	Lower	Upper	RV	Lower	Upper
1. Supine to affected side lateral	-0.04	-0.160	0.071	0.13	-0.05	0.31	0.00	0.0000	<0.0001
2. Supine to non affected side lateral	-0.12	-0.247	0.007	0.07	-0.21	0.35	0.00	0.0000	<0.0001
3. Supine to sitting up on edge of bed	0.05	-0.028	0.135	0.17	-0.05	0.38	0.00	0.0000	<0.0001
4. Sitting without support	0.04	-0.025	0.096	0.17	-0.05	0.38	0.00	0.0000	<0.0001
5. Sitting to standing up	-0.04	-0.128	0.039	0.08	-0.07	0.22	0.00	0.0000	<0.0001
6. Standing with support	-0.07	-0.156	0.022	0.00	-0.17	0.17	0.00	0.0000	<0.0001
7. Standing without support	-0.07	-0.197	0.064	0.00	0.00	<0.01	0.00	<0.0001	0.0131
8. Standing on non paretic leg	0.05	-0.117	0.215	0.04	-0.21	0.28	0.01	<0.0001	0.0374
9. Standning on paretic leg	-0.16	-0.317	0.003	0.05	-0.23	0.33	0.00	0.0000	<0.0001
10. Standing, picking up a shoe from the floor	-0.07	-0.146	0.013	0.00	-0.23	0.23	0.00	0.0000	<0.0001
11. Sitting down from standing up	0.00	-0.064	0.064	0.00	-0.22	0.22	0.00	<0.0001	0.0131
12. Sitting on edge of bed to supine	-0.07	-0.166	0.024	-0.01	-0.24	0.22	0.00	0.0000	<0.0001

CI: Confidence Interval

## Discussion

The aim of this validation study was to investigate the reliability of the modified PASS, SwePASS, in patients with acute stroke. The results indicate both high intrarater and interrater reliability of the scale.

In the study by Benaim *et al *[4], where 12 patients were included to test the intrarater reliability properties of the PASS, six k values were lower than the smallest k value (0.67) in the present study. In contrast, SwePASS with highest k value of 0.93 (item 2 "Supine to affected side lateral"), had no item with a k value of 1.0 as Benaim *et al *[4] showed for items 3 "Supine to sitting up on edge of table" and 9 "Standing on paretic leg". One explanation for these different Kappa values could be the different sample sizes and time span between the assessments. Comparisons to three other reliability studies using the PASS [5-7] are insignificant since the methodology for statistical analysis differed from the analyses applied in the current study.

For items 7 "Standing with support" and 10 "Standing, picking up a shoe from the floor", modifications and specifications in the SwePASS's manual were made. When the score distributions were symmetric and k values were applicable, the present study showed higher k-values, 0.88 compared to 0.76 for "Standing with support" and 0.89 compared to 0.87 for "Standing, picking up a shoe", compared to the results from the intrarater reliability study by Benaim *et al *[4]. This may indicate that the modifications and clarifications in the SwePASS were improvements.

Using the rank-invariant method, described by Svensson [16], for the intrarater reliability, RP, RC and RV also indicate that the SwePASS is a reliable clinical test. The only statistically significant differences that were found, for item 1 and 7, were small. In addition, the rank-invariant method [16], when used in the interrater reliability study, showed no systematic differences between raters and no systematic differences in concentrations of score chosen and only a small non-systematic random variance for item 8. The results for items 1, 3 and 4, which diverged with higher RC, were however statistically non-significant and should be interpreted with caution. Further studies with larger populations are needed to make conclusions about items 1, 3 and 4 regarding RC, items 2 and 9 (which differentiated by higher RC compared to the other items) and item 8 regarding the small RV.

As very few of the patients received score 0 in items 1, 5, 11 and 12 and none among the patients received score 0 in items 2 and 3, the criteria for these items should possibly be changed. A related change of criteria in the PASS has been presented by Wang *et al *[18]. Wang *et al *[18], in a study of 77 stroke patients (mean age of 59.8 years) with a median BBS score of 46, who collapsed the two levels in the centre of the PASS to a single level and recorded each item as 0-1.5-3 and found excellent agreement, Intra Class Correlation ≥0.97, between the new version, called PASS 3P, and the original PASS with four levels. Chien *et al *[9,19] developed a short form of the PASS with 5 items and a 3-level scale. Also Chien *et al *[19], merged the two middle scoring criteria to 1.5, signifying "can perform the activity with help", while score 3 was defined as "can perform the activity without help". This short form of PASS was found to be psychometrically sound, although the floor effect remained [18].

E Svensson's method [16] was chosen because it is specifically developed to make calculations based on paired ordinal data. Ordinal data miss information about size and distance; hence calculations of differences are not appropriate using the more classical methods (t-test, McNemar's test, sign test etc). Some of these classical methods are applicable for ordinal data, but require dichotomization of the data (change toward higher or lower categories on the scale), which means that information from the other categories is missed. This loss of data will not occur when using E Svensson's method, which will use all the data information. Furthermore, E Svensson's method makes it possible to identify and measure systematic disagreement related to the group, when present, separately from disagreement caused by individual variability in assessments. In addition, for further analysis of psychometric properties using the SwePASS, to see whether adding item scores is valid and whether the items in the SwePASS could be reduced without affecting the purpose, an alternative method could be to use the RASCH model [20].

In the current study, a floor effect was found in items 8 and 9, "Standing on the non paretic leg" and "Standing on the paretic leg", in which the majority of the patients were unable to perform the task (score 0). Similar findings have been presented by Benaim *et al *[4] on day 30 after stroke, where score 0 was received by 67% in item 9 and by 43% in item 8. However, the second difficult item in the study of Benaim *et al *[4] was item 10 with 57% of the patients receiving score 0. This relatively large difference in outcomes between studies may be explained by the modification in the current study where a shoe instead of a pencil was used. In case of affected fine motor skills, with inability to pick up the pencil, patients cannot receive any score in this item even if having the ability to change positions. To pick up a shoe, we believe, is less demanding regarding fine motor skills, with less impact on the result.

The opposite, a ceiling effect, was shown in the present study, as many of the patients received the maximum score in many of the items, particularly in items 1 "Supine to affected side lateral" and 4 "Sitting without support". In the study of Benaim *et al *[4] at day 30 after stroke, similar findings were seen with 81% of the patients receiving the score 3, the highest level, in item 4. In addition, on day 90 after stroke, nearly 40% of the patients were scored 36/36.

As could be expected, a higher PA was noted for the intrarater reliability compared to the interrater reliability for each item. Regarding PA (and k) the most difficult items, items 8 ("Standing on non paretic leg") and 9 ("Standing on paretic leg) differed in intrarater reliability measures, (as well as in interrater reliability measures) from the other items. For both the intrarater and the interrater reliability the lowest PA was found in item 8 and 9, which both are of particular importance in hemiplegic patients because monopedal stance is a basic point for the achievement of independent walking. In the intrarater reliability study, 15 out of 20 patients at item 8 and 11 out of 16 patients at item 9 received a higher score at the second test occasion. This could be explained by functional improvement. However, with a relatively short time from test occasion one to two, it seems to be probable that the patient's different approach to the task may have influenced the reliability. Perhaps the patients performed better the second time due to better self-confidence explained by knowing the task and by better awareness of their own performance. Maybe this improvement reflects a practice effect. The size of a possible practice effect might have been smaller; at least theoretically, if the time span between the test occasions had been longer than 24 hours. Still, the time span, one to 24 hours, was chosen to minimize the possible effects of spontaneous recovery.

To our knowledge, no information on average time needed to complete the PASS has been published. However, the previously stated time to complete the PASS "from 1 to 10 minutes depending on the severity of deficits" [4] seems comparable with our average time of eight minutes to perform the SwePASS.

One limitation is the small sample size in the interrater reliability assessments, which would benefit from a reassessment in a larger population. At the time for the intrarater reliability study it was not feasible to perform a large interrater reliability investigation, even if this would had been of great interest. Another limitation, in the intrarater reliability assessments, is the number of raters, who were several. However, the strength is that all the raters were instructed by one of the authors, before the study. Additional strength is the large sample size for the intrarater reliability.

## Conclusions

In conclusion, the modified PASS, SwePASS, showed high intrarater and interrater reliability with both traditional and newer statistical analysis in the acute stage after stroke, particularly for assessments performed by the same rater. These results support the implication for using the SwePASS in the acute stage after stroke, both in clinical and research settings. In addition, the SwePASS was easy to apply and fast to administer in clinic.

## Competing interests

The authors declare that they have no competing interests.

## Authors' contributions

All authors have made substantial contributions to the manuscript. KSS was primary responsible, and CUP and POH was involved, in the conception and design. CUP was primary responsible in the collection, statistical analysis and interpretation of data and in drafting the manuscript. POH, AD and KSS were involved in the interpretation and statistical analysis of data and in drafting. POH, AD together with KSS, who was primarily responsible, revised the manuscript critically for important intellectual content. All authors have read and approved the final manuscript.

## Supplementary Material

Additional file 1 **The Swedish Version of PASS, SwePASS**. The manual for using the SwePASS. Click here for file
