# Supplementary material for: A validation study using a modified version of Postural Assessment Scale for Stroke Patients: Postural Stroke Study in Gothenburg (POSTGOT)
Source: J Neuroeng Rehabil. 2011 Oct 6;8:57. doi: 10.1186/1743-0003-8-57 (PMC3203036; doi:10.1186/1743-0003-8-57)
Supplement: Additional file 1 — The Swedish Version of PASS, SwePASS. The manual for using the SwePASS. [file 1743-0003-8-57-S1.PDF]

The Swedish Version of PASS, SwePASS

Item	Scale	Item	Scale
**1. Supine to affected side lateral**		**7. Standing without support**	
Cannot perform the activity	**0**	Cannot stand without support	**0**
Can perform the activity with support from 2 persons	**1**	Can stand without support for 10 seconds or leans heavily on 1 leg	**1**
Can perform the activity with support from 1 person	**2**	Can stand without support for more than 1 minute or stands slightly asymmetrically	**2**
Can perform the activity without any help	**3**	Can stand without support for more than 1 minute and at the same time draw hand/s from forehand to neck (like drawing finger throw the hair) altered with arm/s hanging parallel with the trunk to avoid tiredness	**3**
**2. Supine to non affected side lateral**		**8. Standing on non paretic leg**	
Cannot perform the activity	**0**	Cannot stand on the non paretic leg	**0**
Can perform the activity with support from 2 persons	**1**	Can stand on the non paretic leg for a few seconds	**1**
Can perform the activity with support from 1 person	**2**	Can stand on the non paretic leg for more than 5 seconds	**2**
Can perform the activity without any help	**3**	Can stand on the non paretic leg for more than 10 seconds	**3**
**3. Supine to sitting up on edge of bed**		**9. Standing on paretic leg**	
Cannot perform the activity	**0**	Cannot stand on the non paretic leg	**0**
Can perform the activity with support from 2 persons	**1**	Can stand on the non paretic leg for a few seconds	**1**
Can perform the activity with support from 1 person	**2**	Can stand on the non paretic leg for more than 5 seconds	**2**
Can perform the activity without any help	**3**	Can stand on the non paretic leg for more than 10 seconds	**3**
**4. Sitting without support**		**10. Standing, picking up a shoe from the floor**	
Cannot sit	**0**	Cannot perform the activity	**0**
Can sit with slight support, for example by 1 hand	**1**	Can perform the activity with support from 2 persons	**1**
Can sit for more than 10 seconds without support	**2**	Can perform the activity with support from 1 person	**2**
Can sit for 5 minutes without support	**3**	Can perform the activity without any help	**3**
**5. Sitting to standing up**		**11. Sitting down from standing up**	
Cannot perform the activity	**0**	Cannot perform the activity	**0**
Can perform the activity with support from 2 persons	**1**	Can perform the activity with support from 2 persons	**1**
Can perform the activity with support from 1 person	**2**	Can perform the activity with support from 1 person	**2**
Can perform the activity without any help	**3**	Can perform the activity without any help	**3**
**6. Standing with support**		**12. Sitting on edge of bed to supine**	
Cannot stand, even with support	**0**	Cannot perform the activity	**0**
Can stand with strong support from 2 persons	**1**	Can perform the activity with support from 2 persons	**1**
Can stand with moderate support from 1 person	**2**	Can perform the activity with support from 1 person	**2**
Can stand with support of only 1 hand	**3**	Can perform the activity without any help	**3**

UNIVERSITAS GOTHOBURGENSIS 1891
UNIVERSITY OF GOTHENBURG

Only one attempt per item is allowed. Ensure that the patient manages the criteria for the scores below, as well as the criterion for the registered score.

A stopwatch is used in items 4 and 7-9 where the patient should maintain a position within a specific time. Item 4 should be performed with the patient´s feet supported on the floor. In items 1-3, 5-6 and 10-12, the patient´s postural balance/control should be scored according to different degrees of support.

SwePASS, developed by Carina Persson, Maria Edvinsson, Katharina Stibrant Sunnerhagen and Ulla Svantesson, published in J Neuroeng Rehabil 2011, is a modified version of the French original version and the published English version of Postural Assessment Scale for Stroke Patients by Benaim C, Pérennou DA, Villy J, Rousseaux M, Pelissier JP. Stroke 1999, 30:1862-1868. Correspondence address: Carina Persson, Institution of Neuroscience and Physiology, Department of Rehabilitation Medicine, Sahlgrenska Academy, University of Gothenburg and Department of Physiotherapy, Sahlgrenska University Hospital/Östra, Göteborg, Sweden carina.persson@vgregion.se
